# Integrated Analysis of the Functions and Prognostic Values of RNA Binding Proteins in Lung Squamous Cell Carcinoma

**DOI:** 10.3389/fgene.2020.00185

**Published:** 2020-03-05

**Authors:** Wei Li, Xue Li, Li-Na Gao, Chong-Ge You

**Affiliations:** Laboratory Medicine Center, Lanzhou University Second Hospital, Lanzhou, China

**Keywords:** lung squamous cell carcinoma, RNA-binding proteins, prognostic signature, survival analysis, bioinformatics

## Abstract

Lung cancer is the leading cause of cancer-related deaths worldwide. Dysregulation of RNA binding proteins (RBPs) has been found in a variety of cancers and is related to oncogenesis and progression. However, the functions of RBPs in lung squamous cell carcinoma (LUSC) remain unclear. In this study, we obtained gene expression data and corresponding clinical information for LUSC from The Cancer Genome Atlas (TCGA) database, identified aberrantly expressed RBPs between tumors and normal tissue, and conducted a series of bioinformatics analyses to explore the expression and prognostic value of these RBPs. A total of 300 aberrantly expressed RBPs were obtained, comprising 59 downregulated and 241 upregulated RBPs. Functional enrichment analysis indicated that the differentially expressed RBPs were mainly associated with mRNA metabolic processes, RNA processing, RNA modification, regulation of translation, the TGF-beta signaling pathway, and the Toll-like receptor signaling pathway. Nine RBP genes (*A1CF, EIF2B5, LSM1, LSM7, MBNL2, RSRC1, TRMU, TTF2*, and *ZCCHC5*) were identified as prognosis-associated hub genes by univariate, least absolute shrinkage and selection operator (LASSO), Kaplan–Meier survival, and multivariate Cox regression analyses, and were used to construct the prognostic model. Further analysis demonstrated that high risk scores for patients were significantly related to poor overall survival according to the model. The area under the time-dependent receiver operator characteristic curve of the prognostic model was 0.712 at 3 years and 0.696 at 5 years. We also developed a nomogram based on nine RBP genes, with internal validation in the TCGA cohort, which showed a favorable predictive efficacy for prognosis in LUSC. Our results provide novel insights into the pathogenesis of LUSC. The nine-RBP gene signature showed predictive value for LUSC prognosis, with potential applications in clinical decision-making and individualized treatment.

## Introduction

Lung cancer is one of the most commonly diagnosed diseases and the leading cause of cancer-related deaths worldwide (Siegel et al., 2019). Lung squamous cell carcinoma (LUSC) accounts for 30% of lung cancer cases, resulting in about 0.4 million deaths each year worldwide (Siegel et al., 2013). Despite advances in diagnosis and treatment of lung cancer over the past few decades, there remains a lack of effective therapies for patients, underscoring the demand for novel treatment methods. Owing to differences in genetic and epigenetic changes among different subtypes of lung cancer, effective treatment targets of adenocarcinoma may not be suitable for LUSC (Wang et al., 2019). Therefore, a systematic study to explore the differentially expressed genes in LUSC is required to identify potential diagnostic markers and therapeutic targets for LUSC.

RNA binding proteins (RBPs) are proteins that interact with various types of RNA and are ubiquitously expressed in cells (Masuda and Kuwano, 2019; New et al., 2019; Otsuka et al., 2019). A total of 1542 RBPs have been identified by high-throughput screening in human cells, representing 7.5% of all protein coding genes (Gerstberger et al., 2014). These RBPs affect post-transcriptional events in cells and modulate cell physiology, and are therefore involved in multiple biological processes including RNA splicing, mRNA stability, export to the cytoplasm, localization, and protein translation (Masuda and Kuwano, 2019; Nahalka, 2019). Given that RPBs perform various critical functions in post-transcriptional events, it is unsurprising that alterations in RBPs are closely related to the initiation and progression of many human diseases. However, the roles of RBPs in the origin and development of cancer remain relatively unexplored.

In recent years, genome-wide analysis has indicated that many RBPs show dysregulated expression in tumors relative to adjacent normal tissues, and that their expression is associated with patient prognosis (Chen et al., 2019; Cooke et al., 2019; Zhang et al., 2019). It is well-known that the dysregulation of RBPs in cancer cells is mainly caused by genomic alterations, microRNA-mediated regulation, epigenetic mechanisms, and post-translational modifications (Gerstberger et al., 2014). Previous studies have linked known cancer drivers to RBP dysregulation. For example, the oncogene *crabp2* interacts with the RBP HuR to promote metastasis of lung cancer cells by regulating integrin β1/FAK/ERK signaling (Wu et al., 2019). Transforming growth factor-β (TGF-β) induces the expression of RNA-binding motif protein 38 (RBM38) in breast cancer, which promotes epithelial-to-mesenchymal transition by regulating the zonula occludens-1 transcript (Wu et al., 2017). The forkhead box K2 protein (FOXK2) promotes colorectal cancer metastasis by upregulating mRNA expression of zinc finger E-box binding homeobox 1 (ZEB1) (Du et al., 2019). Taken together, these studies indicate that the RBPs are closely related to the occurrence and development of human tumors. However, only a small fraction of RBPs have been studied intensively and found to have key roles in cancers to date. Therefore, we collected all relevant LUSC data from The Cancer Genome Atlas (TCGA) database and performed the present systematic analysis to examine the potential molecular functions and clinical significance of RBPs in LUSC. We identified multiple differentially expressed RBPs associated with LUSC, which provide new insight into the pathogenesis of the disease. Some of them may serve as potential biomarkers for diagnosis and prognosis.

## Materials and Methods

### Data Preprocessing and Identification of Differentially Expressed RBPs

RNA sequencing data of 501 LUSC samples and 49 normal lung tissue samples with corresponding clinical information were downloaded from TCGA.^1^ The raw data were preprocessed using the DESeq2 package.^2^ Differentially expressed RBPs were identified based on a false discovery rate < 0.05 and |log_2_ fold change (FC)| ≥ 1. All differentially expressed RBPs had an average count value more than 1.

### GO and KEGG Functional Enrichment Analyses

The biological functions of these differently expressed RBPs were systematically investigated by gene ontology (GO) enrichment, which comprised three terms: molecular function, biological process, and cellular component. The Kyoto Encyclopedia of Genes and Genomes database (KEGG) was used to detect potential biological pathways of differentially expressed RBPs. All GO and KEGG pathway enrichment analyses were carried out using the WebGestalt (WEB-based Gene SeT AnaLysis Toolkit^3^) (Liao et al., 2019) with a *P*-value less than 0.05 and gene number more than 5.

### Protein–Protein Interaction Network Construction and Module Screening

The protein–protein interactions (PPIs) among all differentially expressed RBPs were detected using the START (Search Tool for the Retrieval of Interacting Genes^4^) (Szklarczyk et al., 2019), and their network was constructed with the Cytoscape 3.7.0 software. Subsequently, the key modules were screened from the PPI network with scores >7 and node counts >5 by using the MCODE (Molecular Complex Detection) plug-in in Cytoscape. The cytoHubba plug-in was used to select hub genes. *P* < 0.05 was considered to indicate a significant difference.

### Hub RBPs Expression Validation and Efficacy Evaluation

The Human Protein Atlas (HPA) database^5^ (Uhlen et al., 2017) was used to detect the expression of 10 hub genes at a translational level. Receiver operating characteristic (ROC) curves were constructed with the GraphPad Prism 7.0 software to calculate the ability to discriminate between normal and tumor tissue.

### Copy-Number Alterations and Mutation Analysis of Hub RBPs

The copy-number alteration and mutation data for all hub RBPs from the PPI network were identified using segmentation analysis and the GISTIC algorithm in cBioPortal^6^ (Gao et al., 2013). Next, we carried out a co-expression analysis of all hub RBPs. Then we constructed a network including all hub genes and the 50 most frequently altered neighbor genes.

### Prognosis-Related RBP Selection

The differentially expressed RBPs were subjected to a univariate Cox regression analysis using the survival package in R. A log-rank test was used to select the significant prognosis-related candidate RBPs, and the least absolute shrinkage and selection operator (LASSO), a widely used machine learning algorithm, was used to further predict the prognostic significance of candidate RBPs (iteration equal 1000) (Jiang et al., 2018). We also used a Kaplan–Meier test to evaluate the prognostic value of each candidate RBP identified by LASSO; the RBPs with *P*-value less than 0.05 were considered to be true prognosis-related RBPs.

### Prognostic Model Construction and Evaluation

Based on the selected prognosis-related RBPs genes, we developed a multivariate Cox proportional hazards regression model to predict the prognosis of LUSC patients (Jiang et al., 2017). In this model, the risk score of each sample was calculated according to the following formula:

$$R\,i\,s\,k\,s\,c\,o\,r\,e=\sum_{i=1}^{n}E\,x\,p\,i\,\beta \,i,$$

where β represents the regression coefficient, and *Exp* represents the gene expression value.

To evaluate the performance of this prognostic model, LUSC patients from the TCGA with a survival time greater than 1 month were divided into low- and high-risk subgroups according to the median risk score, and the difference in overall survival (OS) between the two subgroups was compared by a log-rank test. Besides, the SurvivalROC R package was used to construct a ROC curve for prognostic performance of this model, and we drew a nomogram plot to forecast the likelihood of OS using the rms R package. Additionally, 69 LUSC patient samples from the GSE73403 dataset^7^ were used as a validation cohort to confirm the predictive value of the prognostic model.

## Results

### Selection of Differentially Expressed RBPs in LUSC

**FIGURE 1:**
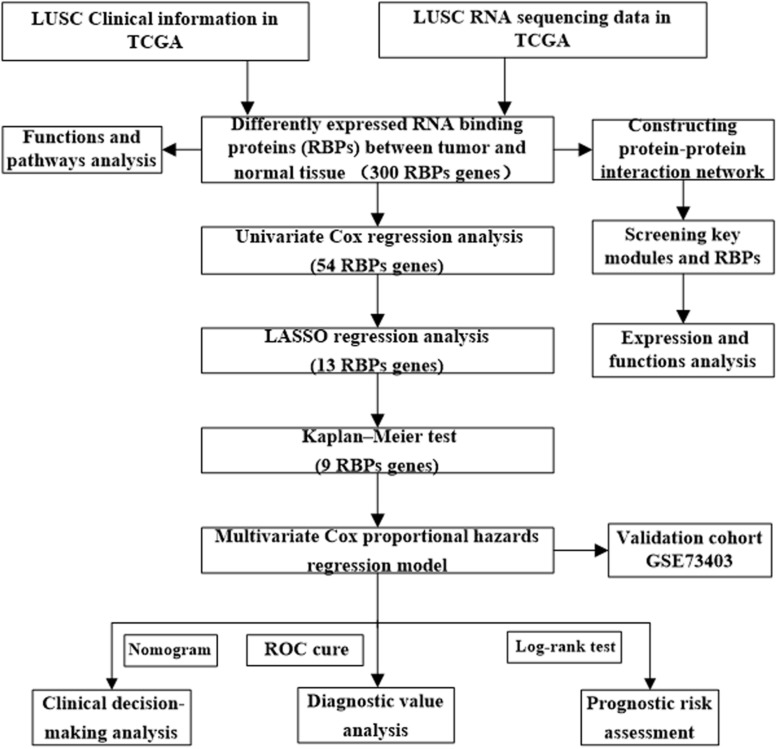
Framework for analyzing the RBPs in LUSC.

The workflow of this study is illustrated in Figure 1. RNA sequencing data for LUSC and corresponding clinical information were downloaded from the TCGA database. A total of 501 LUSC samples and 49 normal lung samples were analyzed. The DESEq2 software packages were used to preprocess these data and detect the differentially expressed RBPs. In total, 1542 RBPs (Gerstberger et al., 2014) were analyzed in this study, of which 300 met our inclusion criteria (adj *P* < 0.05, |log_2_FC| ≥ 1.0), comprising 59 downregulated and 241 upregulated RBPs. The expression distribution of these differentially expressed RBPs is shown in Supplementary Figure S1.

### Functional Enrichment Analysis of the Differentially Expressed RBPs

To explore the potential functional and molecular mechanisms of the identified RBPs, they were divided into two groups based on their expression level. Then we carried out GO and pathway analysis for these differentially expressed RBPs using the online tool WebGestalt. Upregulated differentially expressed RBPs were significantly enriched in biological processes associated with the cellular amide metabolic process, RNA processing, RNA metabolic process, RNA modification, and ribonucleoprotein complex biogenesis (Table 1). The downregulated differentially expressed RBPs were notably enriched in the mRNA metabolic process, RNA processing, defense response to virus, and regulation of translation (Table 1). The molecular function analysis showed that, among the differentially expressed RBPs, the upregulated RBPs were significantly enriched in RNA binding catalytic activity, acting on RNA, structural constituent of ribosome, and nuclease activity (Table 1), whereas the downregulated RBPs were significantly enriched in RNA binding, poly-pyrimidine tract binding, and translation regulator activity (Table 1). In regard to the cellular component, the upregulated RBPs were mainly enriched in the nucleolus, mitochondrial matrix, Sm-like protein family complex, and ribonucleoprotein complex, and downregulated RBPs were mainly enriched in the ribonucleoprotein complex, endolysosome membrane, and RNA cap binding complex (Table 1). Moreover, we found that downregulated differentially expressed RBPs were mainly enriched in the TGF-beta signaling pathway, Toll-like receptor signaling pathway, and mRNA surveillance pathway, whereas upregulated RBPs were significantly enriched for RNA degradation, ribosome biogenesis in eukaryotes, mRNA surveillance pathway, and the spliceosome (Table 1).

**TABLE 1:** GO and KEGG pathway analysis results for differentially expressed RBPs.

**Expression**	**GO term**	***P-*value**
**Up-regulated RBPs**		
Biological processes	Cellular amide metabolic process	<0.001
	RNA processing	<0.001
	ncRNA metabolic process	<0.001
	RNA modification	<0.001
	Ribonucleoprotein complex biogenesis	<0.001
Molecular function	RNA binding	<0.001
	Catalytic activity, acting on RNA	<0.001
	Structural constituent of ribosome	8.34e-14
	Nuclease activity	1.33e-9
Cellular component	Nucleolus	<0.001
	Mitochondrial matrix	<0.001
	Sm-like protein family complex	<0.001
	Ribonucleoprotein complex	<0.001
KEGG pathway	RNA degradation	2.34e-12
	Ribosome biogenesis in eukaryotes	1.27e-11
	mRNA surveillance pathway	2.33e-10
	Spliceosome	<0.001
**Down-regulated RBPs**		
Biological processes	mRNA metabolic process	5.93e-10
	RNA processing	3.31e-7
	Defense response to virus	3.57-7
	Regulation of translation	7.47e-7
Molecular function	RNA binding	<0.001
	Poly-pyrimidine tract binding	5.66e-8
	Translation regulator activity	4.28e-8
Cellular component	Ribonucleoprotein complex	7.9078e-8
	Endolysosome membrane	0.0000033273
	RNA cap binding complex	0.0000041529
KEGG pathway	TGF-beta signaling pathway	0.001
	Toll-like receptor signaling pathway	0.0018
	mRNA surveillance pathway	0.02

### PPI Network Construction and Key Module Screening

**FIGURE 2:**
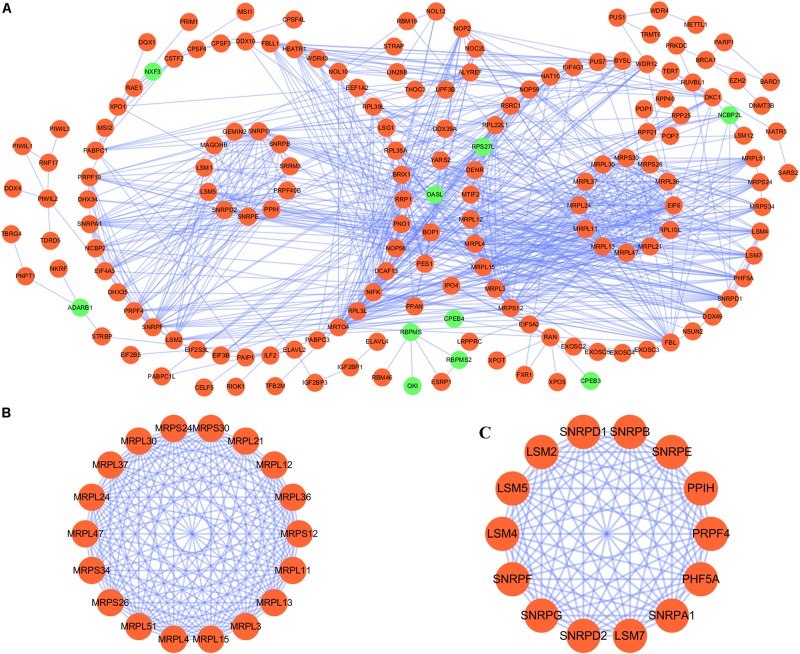
PPI network and module analysis. **(A)** PPI network for RBPs; **(B)** critical module 1 in PPI network; **(C)** critical module 2 in PPI network.

We constructed a protein–protein co-expression network using Cytoscape software and the STRING database, in order to better understand the potential molecular functions of these differentially expressed RBPs in LUSC. This PPI network contained a total of 167 nodes and 771 edges (Figure 2A). Then we screened the hub genes by computing degree and betweenness, and obtained 10 candidate genes*: MRPL15, MRPL13, MRPL4, MRPL3, MRPL24, MRPS12, MRPL11, MRPL21, MRPL36*, and *MRPL47.* Subsequently, we further analyzed the co-expression network to detect potential critical modules by using the plug-in MODE in Cytoscape, and determined the top two significant modules. Module 1 included 18 nodes and 147 edges (Figure 2B), and module 2 consisted of 14 nodes and 91 edges (Figure 2C). The GO and pathway analyses showed that the genes from module 1 were mainly enriched in mitochondrial translation, mitochondrial gene expression, and cellular protein complex disassembly, whereas the genes in module 2 were significantly enriched in spliceosomal snRNP assembly, mRNA splicing, mRNA metabolic process, and RNA processing.

### Hub Gene Expression Validation

**FIGURE 3:**
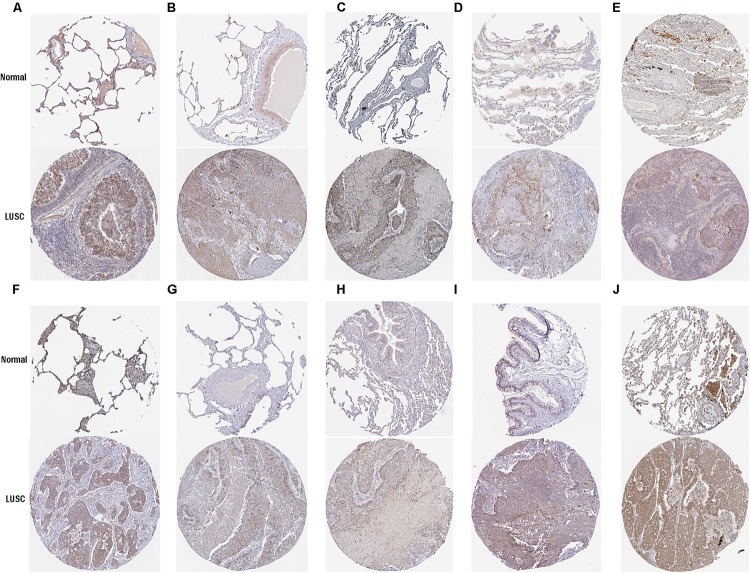
Validation of protein expression of hub genes in normal lung tissue and LUSC using the HPA database. **(A)** MRPL15; **(B)** MRPL13; **(C)** MRPL4; **(D)** MRPL3; **(E)** MRPL24; **(F)** MRPS12; **(G)** MRPL11; **(H)** MRPL21; **(I)** MRPL36; **(J)** MRPL47.

**FIGURE 4:**
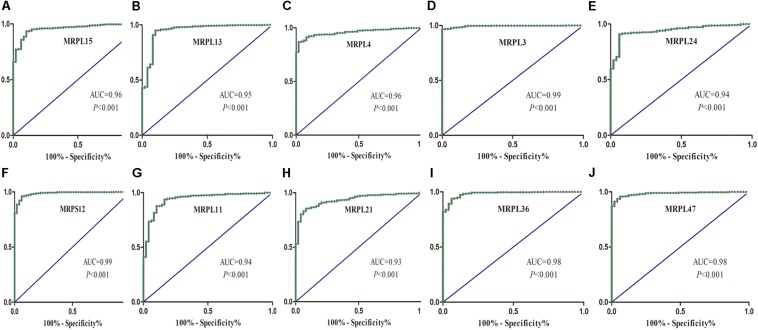
ROC analysis of 10 hub RBPs based on the TCGA dataset. **(A)** MRPL15; **(B)** MRPL13; **(C)** MRPL4; **(D)** MRPL3; **(E)** MRPL24; **(F)** MRPS12; **(G)** MRPL11; **(H)** MRPL21; **(I)** MRPL36; **(J)** MRPL47.

To further determine the expression of these hub genes in LUSC, we used immunohistochemistry results from the Human Protein Atlas database to show that MRPL15, MRPL13, MRPL4, MRPL3, MRPL24, MRPS12, MRPL11, MRPL21, MRPL36, and MRPL47 were significantly increased in lung cancer compared with normal lung tissue (Figure 3). Furthermore, we used ROC curve analysis to evaluate the efficacy of 10 hub genes to discriminate between carcinoma tissue and normal lung tissue. The area under the curve (AUC) of hub genes MRPL15 (AUC = 0.9585, 95% CI: 0.9376–0.9795, *P* < 0.0001), MRPL13 (AUC = 0.9480, 95% CI: 0.9111–0.9849, *P* < 0.0001), MRPL4 (AUC = 0.9578, 95% CI: 0.9407–0.9749, *P* < 0.0001), MRPL3 (AUC = 0.9943, 95% CI: 0.9896–0.9991, *P* < 0.0001), MRPL24 (AUC = 0.9415, 95% CI: 0.9158–0.9672, *P* < 0.0001), MRPS12 (AUC = 0.9862, 95% CI: 0.9758–0.9966, *P* < 0.0001), MRPL11 (AUC = 0.9393, 95% CI: 0.9062–0.9724, *P* < 0.0001), MRPL21 (AUC = 0.934, 95% CI: 0.9074–0.9608, *P* < 0.0001), MRPL36 (AUC = 0.9835, 95% CI: 0.9718–0.9953, *P* < 0.0001), and MRPL47 (AUC = 0.9845, 95% CI: 0.9751–0.9939, *P* < 0.0001) were all greater than 0.9, indicating that the hub genes had higher diagnostic accuracy for LUSC (Figure 4).

### Mutation and Copy-Number Alteration Analysis of Candidate Hub Genes in LUSC Patients

**FIGURE 5:**
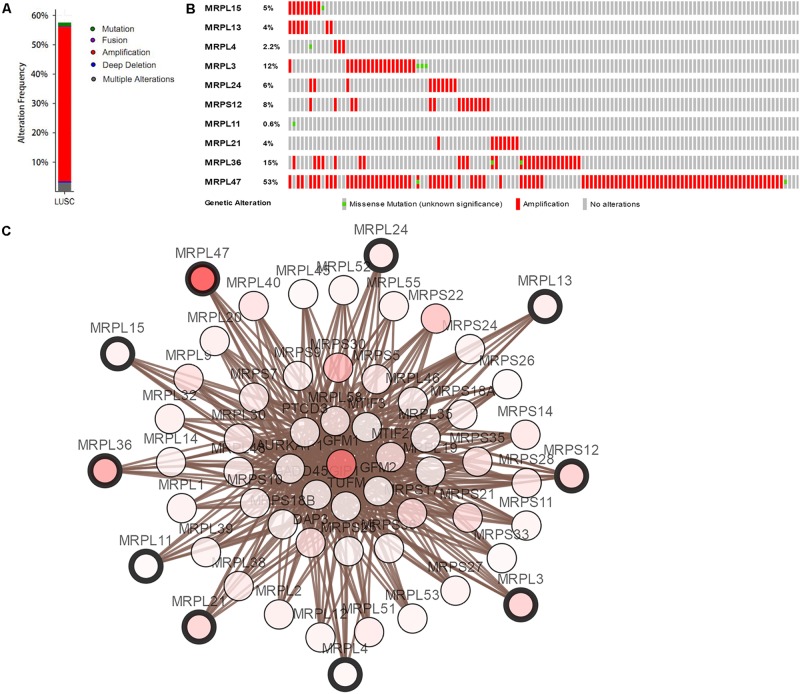
Hub RBP expression and alteration analysis in LUSC. **(A)** Mutation frequency of hub genes; **(B)** mutation frequency of each gene; **(C)** interaction network.

Mutation and copy-number alteration (CNA) analyses of the hub genes MRPL15, MRPL13, MRPL4, MRPL3, MRPL24, MRPS12, MRPL11, MRPL21, MRPL36, and MRPL47 were carried out using the cBioPortal online tool for LUSC (TCGA, Provisional). The results showed that these 10 hub genes were altered in 178 samples out of 511 LUSC patients (35%). Two or more alterations were found in 68% of the LUSC samples (121 samples) (Figures 5A,B). The amplification of *MRPL47* was the most frequent copy-number alteration among these 10 hub genes. Then we constructed an interaction network containing 60 nodes, which comprised 10 hub genes and the 50 most frequently altered neighbor genes (Figure 5C). We also found that mitochondrial translation-related genes, including *GFM1, MTIF2, MTRF1, MRPS10, MRPS11, MRPL1, MRPL9*, and *PTCD3*, were closely associated with alterations of the 10 hub genes.

### Prognosis-Related RBP Screening

**FIGURE 6:**
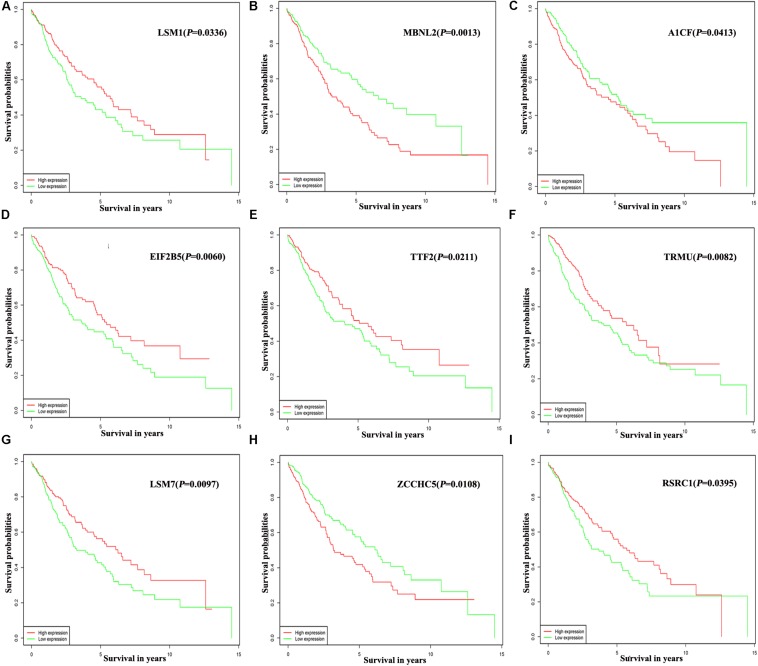
Prognostic value of key nine RBPs in LUSC. **(A)** LSM1; **(B)** MBNL2; **(C)** A1CF; **(D)** EIF2B5; **(E)** TTF2; **(F)** TRMU; **(G)** LSM7; **(H)** ZCCHC5; **(I)** RSRC1.

Of the 300 differentially expressed RBPs, 54 were associated with prognosis as confirmed by univariate Cox regression analysis (Supplementary Table S1). Then we conducted a LASSO regression analysis to obtain the RBP genes with the best potential prognostic significance; 13 RBP genes, *A1CF*, *F4*, *DQX1*, *EIF2B5*, *GEMIN2*, *LSM1*, *LSM7*, *MBNL2*, *PABPC3*, *RSRC1*, *TRMU*, *TTF2*, and *ZCCHC5*, were selected (Supplementary Figure S2). To further determine the RBPs with the greatest potential prognosis ability, a Kaplan–Meier test for OS was used to identify nine RBP-coding genes, *A1CF*, *EIF2B5*, *LSM1*, *LSM7*, *MBNL2*, *RSRC1*, *TRMU*, *TTF2*, and *ZCCHC5* (Figure 6).

### Prognosis-Related Genetic Risk Score Model Construction and Validation

The nine RBPs were analyzed by multiple stepwise Cox regression to construct a predictive model (Table 2). The risk score of each LUSC patient was computed according to the following formula:

**TABLE 2:** Multivariate Cox regression analysis to identify prognosis-related hub RBPs.

**Gene**	**Coef**	**Exp (coef)**	**Se (coef)**	***z***	***Pr*(>| z|)**
LSM1	–0.0134	0.9867	0.0062	–2.1820	0.0291
MBNL2	0.0218	1.0220	0.0114	1.9120	0.0558
A1CF	2.4069	11.0993	1.8701	1.2870	0.1981
EIF2B5	–0.0067	0.9933	0.0054	–1.2340	0.2171
TTF2	–0.0550	0.9465	0.0513	–1.0710	0.2844
TRMU	–0.0557	0.9458	0.0532	–1.0460	0.2955
LSM7	–0.0066	0.9934	0.0079	–0.8410	0.4004
ZCCHC5	1.3639	3.9115	1.7583	0.7760	0.4379
RSRC1	–0.0132	0.9869	0.0223	–0.5920	0.5536

R⁢i⁢s⁢k⁢s⁢c⁢o⁢r⁢e=(0.0218*ExpMBNL2)+(-0.0134*ExpLSM1)+(2.4069*ExpA1CF)+(-0.0067*ExpEIF2B5)

+(-0.0550*ExpTTF2)+(-0.0557*ExpTRMU)

+(-0.0066*ExpLSM7)+(1.3639*ExpZCCHC5)

+(-0.0132*ExpRSRC1)

**FIGURE 7:**
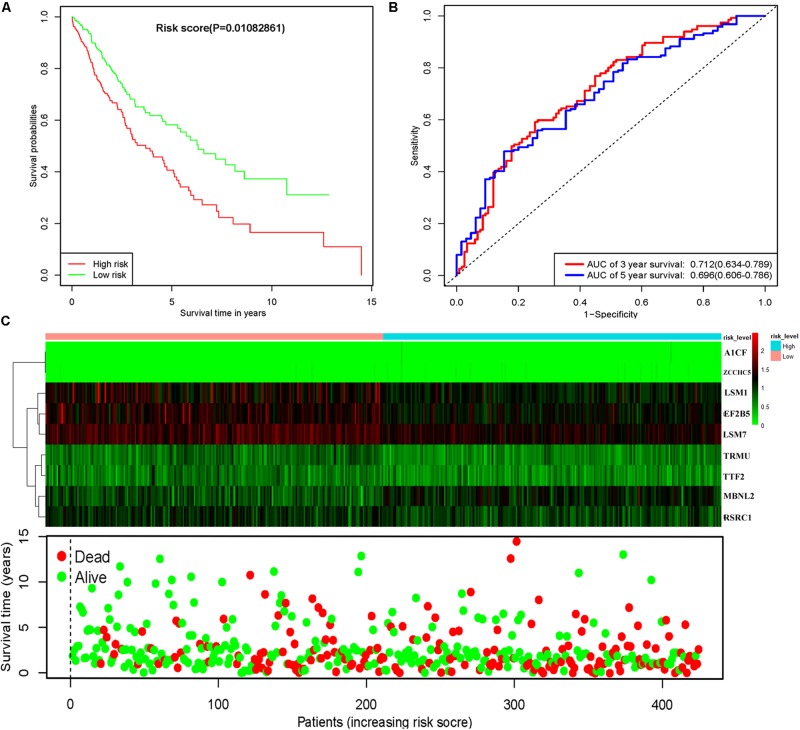
Risk score analysis of nine-gene prognostic model in TCGA LUSC cohort. **(A)** Survival analysis according to risk score; **(B)** ROC analysis; **(C)** heat map and survival status of patients.

**FIGURE 8:**
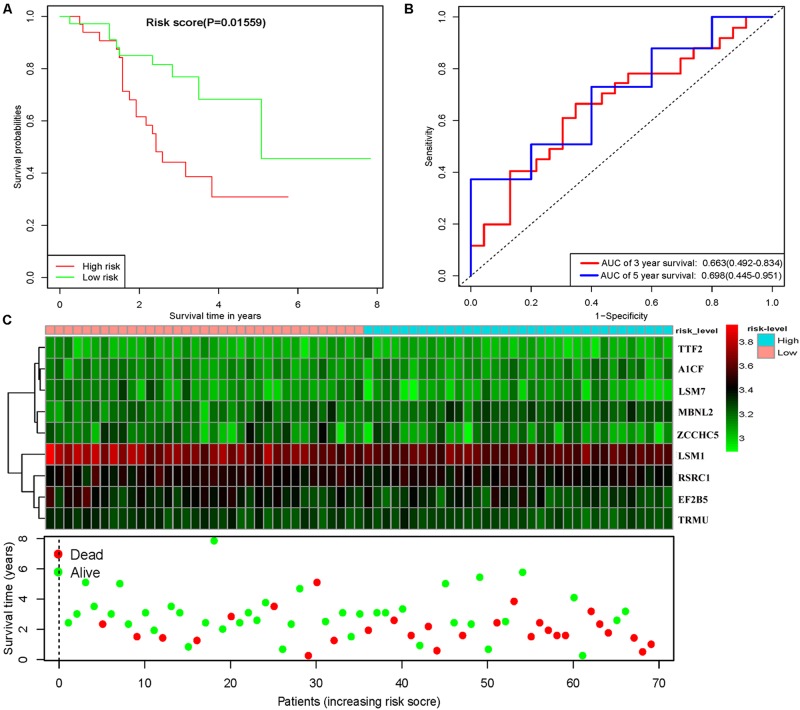
Risk score analysis of nine-gene prognostic model in GSE73403 LUSC cohort. **(A)** Survival analysis according to risk score; **(B)** ROC analysis; **(C)** heat map and survival status of patients.

To assess the predictive ability of this model, we divided 424 LUSC patients into high- and low-risk groups for survival analysis according to the median risk score. Patients in the high-risk subgroup had a significantly lower OS rate than those in the low-risk subgroup (Figure 7A). Then we performed a time-dependent ROC analysis to further evaluate the prognostic performance of the nine-RBP gene signature; the AUC of the ROC curve for OS was 0.712 at 3 years and 0.696 at 5 years (Figure 7B). The expression heat map and survival status of patients with the nine-RBP gene biomarker in the low- and high-risk subgroups are shown in Figure 7C. These results reveal that our prognostic model had moderate sensitivity and specificity. Furthermore, we assessed whether the nine-RBP gene signature predictive model has similar prognostic ability in other LUSC patient cohorts; the same risk assessment formula was utilized to the GSE73403 datasets. The results indicated that patients with high-risk score had poorer OS than those with low-risk score in the GSE73403 cohorts (Figures 8A–C).

**FIGURE 9:**
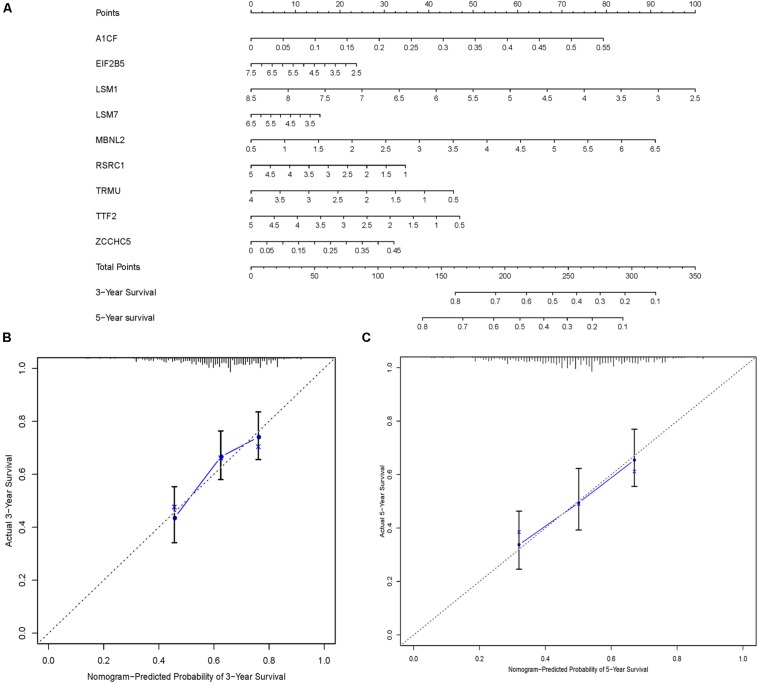
Nomogram and calibration plots of nine RBPs. **(A)** Nomogram to predict 3- and 5-year OS in the TCGA cohort. **(B,C)** Calibration plots of the nomogram to predict OS at 3 and 5 years.

In order to construct a quantitative model for LUSC prognosis, we combined the nine-RBP marker to build a nomogram plot (Figure 9A). This allowed us to calculate the estimated survival probabilities of LUSC patients at 3 and 5 years by plotting a vertical line between the total point axis and each prognosis axis. We constructed calibration plots, which showed that there was good conformity between the predicted and observed outcomes (Figures 9B,C). We also calculated the concordance index for OS in the TCGA and GSE16011 cohorts, which were 0.69 and 0.66 respectively. In addition, we evaluated the prognostic value of different clinical features in 335 patients with LUSC by conducting a univariate regression analysis. The results indicated that age, smoking, stage, distant metastasis, and risk score were related to OS of LUSC patients (*P* < 0.01) (Table 3). However, we only found that age, smoking, and risk score were independent prognostic factors related to OS through multiple regression analysis (Table 3).

**TABLE 3:** The prognostic effect of different clinical parameters.

	**Univariate analysis**			**Multivariate analysis**		
		
	**HR**	**95% CI**	***P*-value**	**HR**	**95%CI**	***P*-value**
Age	1.03	1.01–1.08	0.003	1.04	1.02–1.08	<0.001
Gender	1.19	0.81–1.75	0.380	1.21	0.82–1.80	0.333
Smoking	0.80	0.67–0.94	0.009	0.73	0.61–0.87	<0.001
Stage	1.24	1.01–1.51	0.036	1.50	0.93–2.41	0.0956
T	1.22	0.99–1.54	0.095	1.02	0.74–1.41	0.910
M	2.74	1.01–7.44	0.049	1.01	0.26–3.99	0.985
N	1.08	0.85–1.37	0.542	0.83	0.53–1.29	0.400
Risk score	1.93	1.55–2.40	<0.001	2.06	1.64–2.59	<0.001

## Discussion

Malignant tumors are characterized by uncontrolled cell growth, which is mainly due to the dysregulated expression of cancer driver genes that regulate cell proliferation and differentiation. This includes gain of function mutations of oncogenes and functional deletion alterations of tumor-suppressor genes, or disabling of genome maintenance genes (Masuda and Kuwano, 2019; Zhou et al., 2019). Many studies have reported that RBPs show dysregulated expression in various human cancers (Dong et al., 2019; Soni et al., 2019; Velasco et al., 2019). However, little is currently known about the expression patterns and roles of RBPs in LUSC. In the present study, we integrated TCGA RNA sequencing data for LUSC and identified differentially expressed RBPs between cancer and normal tissue. We systematically investigated relevant biological pathways and constructed PPIs for these RBPs. Then, we performed survival analyses, ROC analyses, and copy-number alterations analyses to explore the potential biological functions and clinical values of the hub RBPs. We also screened key prognosis-related RBPs and constructed a risk model to predict LUSC prognosis based on a nine-RBP gene signature.

The biological functions and pathway enrichment analysis of these differentially expressed RBPs showed that the upregulated RBPs were significantly enriched in the cellular amide metabolic process, RNA processing, RNA metabolic process, RNA modification, RNA degradation, ribosome biogenesis, and mRNA surveillance pathway. The downregulated RBPs were mainly enriched in the mRNA metabolic process, RNA processing, regulation of translation, TGF-beta signaling pathway, and Toll-like receptor signaling pathway. In recent years, a large number of studies, have reported the role of aberrant RNA metabolism and RNA processing in various diseases (Li et al., 2017, 2018; Li S. et al., 2019; Li Y. et al., 2019). RNA processing factors were shown to have increased expression in poorly differentiated non-small-cell lung cancer cells (Geles et al., 2016). The TGF-beta signaling pathway is a classical tumorigenesis-related pathway; it exerts dual and opposing roles in oncogenesis, inhibiting cell proliferation in early tumors and inducing tumor progression and metastasis in advanced cancer (Seoane and Gomis, 2017; Batlle and Massague, 2019). Previous studies have shown that RBPs can interact with the TGF-beta signaling pathway to regulate lung carcinogenesis (Kim et al., 2016; Bai et al., 2019). These results suggest that RBPs can affect the growth of tumor cells by regulating multiple biological processes, such as the TGF-beta signaling pathway, RNA metabolism, and RNA processing.

Subsequently, we obtained 10 hub RBPs by constructing a PPI network: MRPL15, MRPL13, MRPL4, MRPL3, MRPL24, MRPS12, MRPL11, MRPL21, MRPL36, and MRPL47. These hub RBPs are mitochondrial ribosomal proteins that are essential for maintaining mitochondrial functions. Impaired mitochondrial functions such as apoptosis and oxidative phosphorylation are found in most cancers, however, their mechanisms are unclear (Koc et al., 2015; Lee et al., 2017; Lin et al., 2019). Lee et al. (2017) found that suppressed MRPL13 expression increased hepatoma cell invasiveness. Koc et al. (2015) proposed that defects in mitochondrial function in head and neck squamous cell carcinoma might be caused by a decrease in MRPL11 expression. Shi et al. (2015) revealed that MRPL21 was significantly overexpressed in esophageal squamous cell carcinoma (ESCC) and could be used as a candidate prognostic biomarker. Although little is known about the relationship between mitochondrial ribosomal proteins and LUSC, our results indicate that impaired mitochondrial function is an important cause of LUSC, and further evaluation of potential roles of the 10 differentially expressed hub mitochondrial ribosomal proteins in LUSC may be worthwhile.

In addition, the prognosis-related hub RBPs were screened using univariate Cox regression analysis, LASSO regression analysis, Kaplan–Meier test, and multiple Cox regression analysis. We finally determined nine RBP-coding genes: *A1CF, EIF2B5, LSM1, LSM7, MBNL2, RSRC1, TRMU, TTF2*, and *ZCCHC5*. High expression of *LSM1, EIF2B5, TTF2, TRMU, LSM7*, and *RSRC1* was associated with a good prognosis in patients with LUSC, whereas that of *A1CF, MBNL2*, and *ZCCHC5* were related to poor prognosis. Next, the nine RBPs were used to construct a risk model by multiple stepwise Cox regression analysis to predict prognosis in LUSC patients. The ROC curve of the prognostic model showed that the nine-RBP genes signature had moderate performance for predicting OS at 3 years (AUC = 0.712) and 5 years (AUC = 0.696). A nomogram was constructed to enable practitioners to predict 3-, and 5-year OS of LUSC patients. According to the outcomes predicted by our model, patients with high risk scores have a poor prognosis, suggesting that they may need an adjusted treatment plan and individualized treatment.

Overall, our study provides novel insights into the role of RBPs in the tumorigenesis and progression of LUSC. Furthermore, our prognostic model showed good predictive performance with regard to survival, which may contribute to the development of new prognostic indicators for LUSC. Furthermore, the RBP-related gene marker showed a pivotal biological background, which demonstrates that these RBPs could be used in clinical adjuvant treatments. Nevertheless, our study had several limitations. First, our results were only based on single-omics (RNA sequencing); patients may exhibit heterogeneity owing to the different features of other omics data platforms. Moreover, our prognostic model was built on the TCGA LUSC dataset and was not validated with a clinical patient cohort; a prospective study should be performed to verify the results. Finally, the lack of some clinical characteristics in the datasets from TCGA may have decreased the statistical effectiveness and reliability of the multivariate stepwise Cox regression analysis.

## Conclusion

We investigated the expression, potential functions, and prognostic values of aberrantly expressed RBPs via a series of bioinformatics analysis in LUSC. These RBPs were associated with oncogenesis, development, invasion, and metastasis. A nine-RBP coding gene prognostic model was developed that could act as an independent prognostic signature for LUSC. To the best of our knowledge, this is the first report of the establishment of an RBP-associated prognostic model for LUSC. These findings provide important insight into the pathogenesis of LUSC, which may contribute to clinical decision-making and individualized treatment.

## Data Availability Statement

The RNA-sequencing data of 501 LUSC samples and 49 normal lung tissue samples with corresponding clinical information were downloaded from the Cancer Genome Atlas (TCGA) database (https://portal.gdc.cancer.gov/).

## Author Contributions

WL and CG-Y conceived and designed the study and wrote the manuscript. WL, XL, and L-NG analyzed the data. All authors reviewed and approved the final manuscript.

## Conflict of Interest

The authors declare that the research was conducted in the absence of any commercial or financial relationships that could be construed as a potential conflict of interest.

## Footnotes

1 https://portal.gdc.cancer.gov/

2 http://www.bioconductor.org/packages/release/bioc/html/DESeq2.html

3 http://www.webgestalt.org/

4 http://www.string-db.org/

5 http://www.proteinatlas.org/

6 https://www.cbioportal.org/

7 https://www.ncbi.nlm.nih.gov/gds/?term=GSE73403
