# Supplementary material for: Integrated Analysis of the Functions and Prognostic Values of RNA Binding Proteins in Lung Squamous Cell Carcinoma
Source: Front Genet. 2020 Mar 5;11:185. doi: 10.3389/fgene.2020.00185 (PMC7066120; doi:10.3389/fgene.2020.00185)
Supplement: Supplementary file 1 [file Presentation_1.pdf]

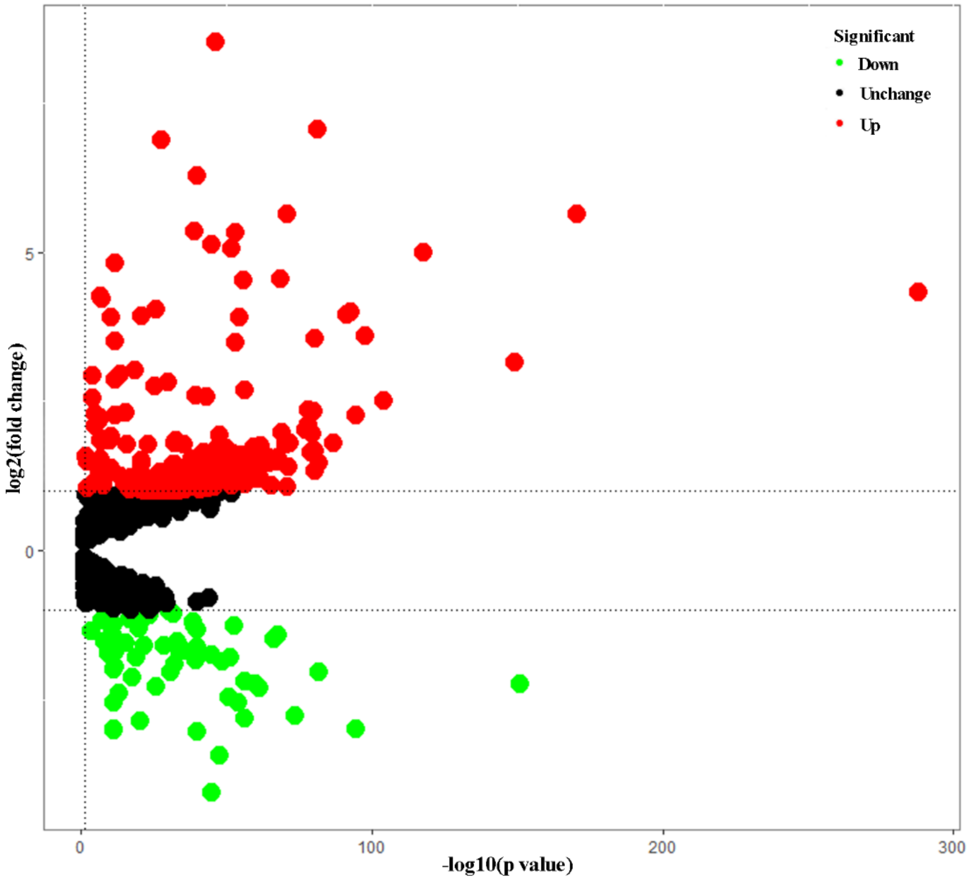

Figure S1 Differentially expressed RBPs in LUSC. Red: upregulation with FC >2; green: downregulation with FC >2; black: not significant.

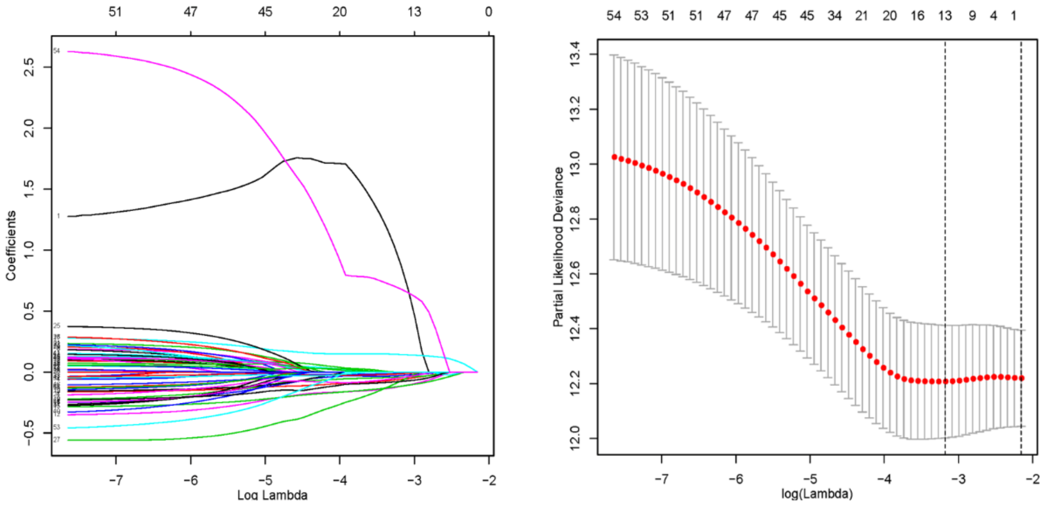

Figure S2 LASSO regression analysis for screening prognosis-related RBPs.
