# Supplementary material for: Integrated Analysis of the Functions and Prognostic Values of RNA Binding Proteins in Lung Squamous Cell Carcinoma
Source: Front Genet. 2020 Mar 5;11:185. doi: 10.3389/fgene.2020.00185 (PMC7066120; doi:10.3389/fgene.2020.00185)
Supplement: Supplementary file 2 [file Table_1.DOCX]

Table S1 Univariate Cox regression analysis to identify prognosis related-RBPs in the TCGA dataset

| **Gene name** | **HR** | ***P* value** | **Gene name** | **HR** | ***P* value** |
| --- | --- | --- | --- | --- | --- |
| MBNL2 | 1.497 | 0.000 | SNRPG | 0.749 | 0.015 |
| SMAD7 | 1.393 | 0.001 | CTIF | 1.396 | 0.016 |
| POLR2H | 0.701 | 0.001 | EZH2 | 0.794 | 0.016 |
| LSM1 | 0.724 | 0.002 | NPM3 | 0.779 | 0.016 |
| LSM7 | 0.665 | 0.002 | TRMT2A | 0.739 | 0.016 |
| CPSF4 | 0.650 | 0.003 | DDX55 | 0.661 | 0.017 |
| ZCCHC5 | 23.493 | 0.004 | NOVA2 | 1.850 | 0.017 |
| DQX1 | 0.768 | 0.004 | RNASE1 | 1.153 | 0.017 |
| TTF2 | 0.653 | 0.004 | EXOSC3 | 0.730 | 0.019 |
| EIF2B5 | 0.722 | 0.004 | PRPF19 | 0.682 | 0.025 |
| RSRC1 | 0.675 | 0.004 | A1CF | 17.067 | 0.026 |
| GEMIN2 | 0.659 | 0.005 | RPL22L1 | 0.839 | 0.027 |
| POP7 | 0.669 | 0.005 | MRPL51 | 0.713 | 0.028 |
| TRMU | 0.647 | 0.005 | TARBP1 | 0.780 | 0.030 |
| TRMT1 | 0.697 | 0.006 | KHDC1 | 0.721 | 0.030 |
| PTCD1 | 0.582 | 0.007 | NOP58 | 0.727 | 0.031 |
| PHF5A | 0.701 | 0.007 | RIOK1 | 0.722 | 0.033 |
| SECISBP2L | 1.519 | 0.008 | UPF3B | 0.766 | 0.035 |
| LSG1 | 0.749 | 0.008 | NIFK | 0.741 | 0.036 |
| CPEB4 | 1.489 | 0.009 | DDX39A | 0.783 | 0.037 |
| RNASEH2A | 0.759 | 0.012 | PABPC3 | 0.505 | 0.039 |
| TLR3 | 1.434 | 0.012 | NOL10 | 0.700 | 0.040 |
| NCBP2 | 0.736 | 0.012 | BRIX1 | 0.775 | 0.040 |
| MRPL47 | 0.759 | 0.013 | ZCCHC24 | 1.205 | 0.041 |
| NOL12 | 0.657 | 0.013 | SF3B6 | 0.726 | 0.042 |
| ZC3H8 | 0.625 | 0.013 | SNRPA1 | 0.727 | 0.044 |
| RPL35A | 0.774 | 0.014 | WDR3 | 0.756 | 0.049 |
